# Marker-Assisted Selection for Recognizing Wheat Mutant Genotypes Carrying HMW Glutenin Alleles Related to Baking Quality

**DOI:** 10.1155/2014/387912

**Published:** 2014-04-27

**Authors:** Mohammad Javad Zamani, Mohammad Reza Bihamta, Behnam Naserian Khiabani, Zahra Tahernezhad, Mohammad Taher Hallajian, Marzieh Varasteh Shamsi

**Affiliations:** ^1^Seed and Plant Improvement Institute, Fahmideh Boulevard, Karaj, Iran; ^2^Agriculture College, Tehran University, Karaj, Iran; ^3^Agricultural, Medical and Industrial Research School, Karaj, Iran; ^4^Agriculture College, Zanjan University, Zanjan, Iran; ^5^Agriculture College, University of Payam Nour, Kaboudarahang Branch, Kaboudarahang, Iran

## Abstract

Allelic diversity of HMW glutenin loci in several studies revealed that allelic combinations affect dough quality. Dx5 + Dy10 subunits are related to good baking quality and Dx2 + Dy12 are related to undesirable baking quality. One of the most regular methods to evaluate the baking quality is SDS-PAGE which is used to improve baking quality labs. Marker-assisted selection is the method which can recognize the alleles related to baking quality and this method is based on polymerase chain reaction. 10 pairs of specific primers related to Dx2, Dx2.1, Dx5, Dy10, and Dy12 subunits were used for recognizing baking quality of some wheat varieties and some mutant genotypes. Only 5 pairs of them could show the specific bands. All subunits were recognized by the primers except Dx2.1. Some of the primers were extracted from previous studies and the others were designed based on D genome subunits of wheat. SDS-PAGE method accomplished having confidence in these marker's results. To realize the effect of mutation, seed storage proteins were measured. It showed that mutation had effect on the amount of seed storage protein on the mutant seeds (which showed polymorphism).

## 1. Introduction

Bread wheat (*Triticum aestivum*) is a kind of allohexaploid which has A, B, and D genomes. High molecular weight glutenin (HMW-Gs) subunits are important combinations of wheat glutenin proteins which play an important role in viscosity and elasticity of wheat dough [1]. Nullisomic, tetrasomic, nulli-tetrasomic, and ditelocentric series lines showed that high molecular weight glutenin are controlled by the loci on long arm of 1A, 1B, and 1D chromosomes near centrometer (recombination index = 9%) [2]. Each locus has two linked genes which are named x and y type with different molecular weight [3].

Bread wheat contains six HMW glutenin subunit-coding genes (two x type and two y type), but silencing of specific genes causes the creation of only three to five HMW protein subunits [4]. The y genes in hexaploid wheat A genome are silent but they are active in several diploid and tetraploid wheat [5].

Glu-A1 locus is distinguished by three main x type alleles which are coded 1, 2*, and null subunits [6]. In several bread wheat and durum wheat, there is Bx7 allele in Glu-B1 locus commonly [7]. The subunits Ax1 and Ax2* HMW glutenin alleles are in Glu-A1, Bx17 + By18, Bx7 + By8, or By9 are in Glu-1B locus, and Dx5 + Dy10 are in Glu-1D locus, related to baking quality, on the other hand, AxNull, Bx6 + By8, and Dx2 + Dy12 are related to undesirable baking quality [5]. SDS-PAGE is one of the most regular techniques which is used in wheat improvement labs to recognize different allelic forms related to baking quality [8]. Wheat breeders selected different variety for baking quality by using SDS tests, Zeleny sedimentation, micrograph assessment, and other methods. These procedures are not useful in initial hybrids because these methods need a large amount of seed, also these methods destroy experimental samples, but improved PCR based on HMW glutenin subunit markers showed baking quality in initial segregation hybrids [9].

Glu-1 loci sequences are recognized by Gu et al. [10], Kong et al. [11], Anderson and Greene [12], Halford et al. [4], and Wan et al. [13].

Many researchers studied different HMW glutenin subunits in wheat cultivars by analysis based on PCR [8, 14–19]. Moczulski and Salmanowicz [16] reported that rapid recognition of Glu-1 genes molecular markers by using multiplex PCR could be standard and effective to select initial useful wheat genotypes.

DNA markers are easy and rapid way for selection. These markers could be replaced as standard and creditable techniques to select genotypes which have HMW glutenin subunits related to baking quality [20].

The goal of this study was recognition of wheat genotypes carrying glutenin allele combinations by using specific primers and PCR methods, studying about the effect of mutation on seed storage proteins, and selecting these mutant genotypes to continue breeding programs.

## 2. Materials and Methods

### 2.1. Plant Material

48 genotypes (mutant lines: Roshan, Tabasi, Azar, Azadi, and Omid with their parents, also some other cultivars like Chamran, Chinese spring and so on) were used. These mutant lines only were supplied.

Tabasi and the mutants of Tabasi: T-66-58-6, T-65-9-IP, T-65-5-1, T-65-6, T-66-58-9, T-66-58-12, T-66-58-10, T-65-9-1, T-65-4, T-58-8, T-67-7-1, T-66-I-II, T-58-7, T-67-60, T-58-14, T-65-9-II-4, and T-66-58-6; Omid and the mutants of Omid: O-64-4, O-64-1-1, and O-6-1-1; Roshan and the mutants of Roshan: Ro-1, Ro-2, Ro-3, Ro-4, Ro-5, Ro-6, Ro-7, Ro-8, Ro-9, Ro-10, Ro-11, and Ro-12; Azadi and the mutants of Azadi: As-48, Azar, Azar mutant, Chamran, Tajan, Tajan-e-garm, Atrak, Navid, Inia, Bezostaya, Pishtaz, and Chinese spring.

### 2.2. DNA Extraction and Polymerase Chain Reaction (PCR)

Genomic DNA of each genotype was extracted from young leaf by CTAB procedure [21]. Quantity and quality of genomic DNA were measured by spectrophotometer in 260 nm and 1% Agarose gel. Polymerase chain reaction was accomplished based on sequence tagged sites (STS) primers which were specified for alleles related to HMW glutenin alleles. The sequences of primers exist in Table 1.

Reaction combinations in 25 *μ*L were 1X PCR buffer, 2 mM MgCl_2_, 5 mM dNTPs, 10 mM of each primer, 1.5 U Taq DNA polymerase, 250 ng of genomic DNA, and ddH_2_O (up to 25 *μ*L), also the PCR program was 94°C for 2′ (initial denaturing) and 30 cycles: 94°C for 1′ (denaturing), 58–66°C for 1′ (annealing), and 70–72°C for 1′ (extension) and the final extension was 72°C for 6′ (Table 2). Amplified products separated by agarose gel 1.7% and staining were by Ethidium Bromide.

### 2.3. SDS-PAGE Method

The HMW glutenin subunits were extracted from the seeds of each accession without embryo, based on modified Laemmli [23], then separated by electrophoresis in polyacrylamide gel (main gel was 10% and stacking gel was 4%). After electrophoresis, the gels were stained by Coomassie Brilliant Blue R250 for 24 hours; the gels were left in TCA 10% for 3-4 hours, then in distilled water for 12 hours. Finally, the gels scanned and analyzed.

## 3. Results and Discussion

### 3.1. PCR and SDS-PAGE

**Figure 1:**
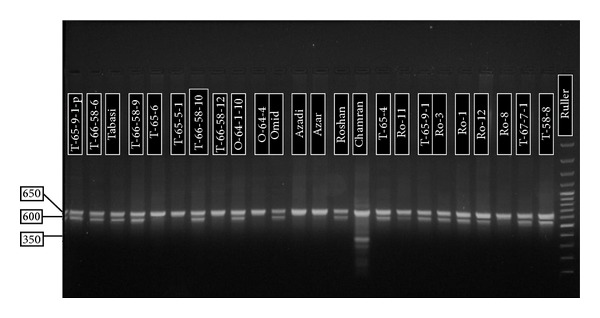
Agarose gel (1.7%) for P1P2 (Dy10 and Dy12).

By P1P2, the genotypes, which have Dy10 and Dy12, showed the band 650 bp. All of the genotypes revealed this band. In addition, two other bands were observed. The first one was 350 bp, only Chamran showed this band, and the other one was 600 bp in Tabasi, Omid, Roshan, and some of the other mutants. The 600 bp band was reported by Smith et al. [22], but 350 bp band was not reported by any researchers (Figure 1). T-66-58-12 genotype is similar to Azadi (both of them have only 650 bp band), but Tabasi and Omid have both 650 bp and 600 bp bands. These results confirm the SDS-PAGE result. For example, Tabasi and Omid have similar banding pattern in STS and also in SDS-PAGE method.

**Figure 2:**
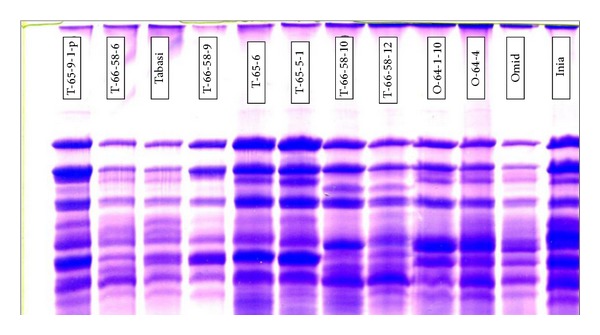
SDS-PAGE polyacrylamide gel for seed storage protein.

The P3P4 primers amplified four kinds of bands in which three of them were reported by Smith et al. [22]. The first one was 576 bp for Dy10 and the second one was 612 bp that belongs to Dy12. These bands according to the sequences of wheat are specific. In addition, Ahmad [8] used these primers and reported two bands which belong to Dy10 and Dy12. Like P1P2, all of the genotypes had one of these alleles (Dy10 or Dy12) also, SDS-PAGE results confirm the result of P1P2 and P3P4 (Figure 2). The third band was 675 bp which belongs to By9; all of the genotypes showed this allele except three mutants (Ro-9, T-65-9-1P, and T-65-9-1). The last band was only in Azar mutant, this band was not reported by Smith et al. [7] and this has occurred because of the mutation.

**Figure 3:**
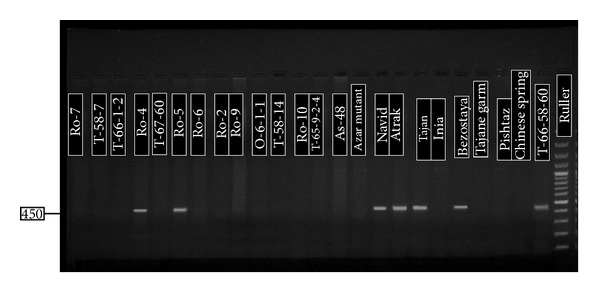
Agarose gel (1.7%) for P5P6 (Dy10 and Dy12).

P5P6 primers were for identifying Dx5 [8]. Only the genotypes that have Dx5 showed the band 450 bp. In this study, 11 genotypes showed this band. Five genotypes were Navid, Atrak, Tajan, Bezostaya, and Chamran, and the other genotypes were the mutants of Tabasi, Omid, and Roshan (Figure 3). Because nonmutant genotypes did not show this band, maybe mutation was the cause of this event. Ahmad [8] used these primers to separate the genotypes that had Dx5 allele from the genotypes which had Dx2 allele. In SDS-PAGE method, because of closeness of Dx2 and Dx5, the recognition is very hard. By PCR method and using these specific primers, the recognition of these alleles is so easy.

P7P8 primers belong to Dx5 like P5P6. According to De Bustos et al. [15], these primers amplify a band in 2775 bp, but in this study there were two bands (550 and 1350 bp) for all genotypes. Therefore these primers cannot be used for marker-assisted selection, because of the absence of specific and polymorphic band.

By using P9P10, primers that amplify Dy10 allele, one single band occurred in eleven genotypes. Five of these genotypes were the controller cultivars (Navid, Atrak, Tajan, Bezostaya, and Chamran) and the others were the mutants of Tabasi, Omid, and Roshan. Therefore, by using these primers, the Dy10 alleles can be recognized.

P11P12 primers were for recognition of Dx2. The genotypes, which had this allele, showed single 2799 bp band. All of the genotypes except eleven (which had Dy10 allele) showed this band.

P13P14 primers were the last primers, which were extracted from the study of De Bustos et al. [15]. They reported a 2190 bp band that belongs to Dy12. However, this band did not appear in spite of changes like combination of PCR materials, PCR cycle, and so forth.

P15P16, P17P18, and P19P20 primers which were for amplifying Dx5 (2520 bp), Dx2.1 (651 bp), and Dy10 (1947 bp) respectively, like the previous primers did not show any specific band appearing in spite of all the changes. Therefore, these primers cannot be used for selection by using marker.

### 3.2. Protein Percentage

Comparing of protein mean with Duncan's multiple-range test showed that there is a significant difference between some mutants and their origin genotypes (Table 3). The Roshan mutant genotypes, which are named Ro-1 and Ro-5, had significantly lower protein percentage in comparison with Roshan, on the other hand, Ro-3 had higher protein percentage than Roshan. There was not any significant difference of protein percentage between Tabasi and Omid with their mutants.

Usually the movement of HMW glutenin subunits in SDS-PAGE method does not have a complete relationship with its molecular weight and this event may cause trouble for breeders to select the lines, as Shewry et al. [7] reported. Goldsbrough et al. [24] reported that a significant difference exists between molecular weight and movement of HMW subunits in SDS-PAGE method. These alleles can be recognized by comparing these HMW glutenin subunits in SDS-PAGE method. Marker-assisted selection is an effective and reliable method which is based on the gene sequence and polymerase chain reaction.

Glutenins are combinations of high molecular weight subunits (HMW) and low molecular weight (LMW) which are named according to the migration ratio in gel [25]. Allelic diversity in each glutenin locus and several studies showed allelic combinations affected dough quality [5]. HMW glutenin subunits 5 + 10 were related to high elasticity and good baking quality; however, 2 + 12 subunits were related to low baking quality [26]. 31% of smooth wheat cultivars have 5 + 10 subunits and many of breeders reported the existence of 5 + 10 in smooth wheat lines [27].

In this study, ten pairs of specific primers were used for recognition of Dx2, Dx2.1, Dx5, Dy10, and Dy12 but only five pair of them showed specific bands. All of the alleles were recognized except Dx2.1, Dy10, and Dy12 were recognized by P1P2 and P3P4 primers. By using P1P2 and P3P4 primers, Smith et al. [22] and Ahmad [8] identified the genotypes that carried Dy10 and Dy12. The results showed all the genotypes had Dy10 or Dy12 allele. P1P2 primers could not separate Dy10. Ahmad [8] reported the coding genes of Dy10 and Dy12 had 98.9% similarity, he found three regions for designing of primers and these three regions were Dy10, Dy12, and Dy9.

The results from amplification of fragments showed a high linkage between Dy12 and Dy10.

Liu et al. [19] amplified the genes related to Glu D1 (x2, x5, y10, and y12) alleles by codominant markers. Also Schwarz et al. [28] used SNP markers for recognition of Dx2 and Dx5 subunits.

HMW glutenin subunits were related to elasticity and baking quality and a large amount of diversity between cultivars is because of HMW glutenin subunits [25]. The sequence of Glu-A1x2* and Glu-D1x5 genes was analyzed and compared by Anderson and Greene [12]. These results showed a high similarity in their structures, therefore separating of the alleles by SDS-PAGE method is hard. In addition, there was a complete conformity between the results of HMW glutenin allelic combination in this study, also D'Ovidio and Anderson [18] reported a complete conformity between PCR and allelic combination of wheat samples in SDS-PAGE method.

High genetic linkage between x and y subunits causes trouble to recognize of subunits that are related to baking quality [18].

## 4. Conclusion

All of the studies in this field emphasize on the importance of this kind of marker to recognize the gene which affects dough characteristics. The method needs only a few amount of leaf DNA and to prevent the injury (by separating of a piece of endosperm) to embryo [14]. By the method (marker-assisted selection), selecting and studying about the plants can be possible in all of the life cycle of plant.

By marker-assisted selection, the recognition between DNA samples which carried alleles related to baking quality is simple and the results could be achieved in about three hours. The method omits using dangerous material like Acrylamide and this method is more reliable. Because of the closeness of x2 and x5 protein coding molecule weight and movement of HMW glutenin subunits in gel some miss scoring occurred. On the other hand, some mutants in x5 cannot be recognized by SDS-PAGE method, therefore marker-assisted selection is a reliable way to select genotypes. Another benefit to this method is that by using any part of the plant (leaf, root, seed, and stem) this selection can be done. Using multiplex, PCR (amplifying some alleles at the same time) can decrease the payment [17]. By marker-assisted selection, more then 1000 plants can be selected and evaluated at the first stage of plant growth by breeders [8]. Using of diploid part of a plant in comparison to endosperm (triploid) is easier and faster in commenting of the results in the heterozygous plants [22]. Finally, the last benefit of this marker is that the marker is not affected by internal or external environment.

## Figures and Tables

**Table 1:** STS primers used to amplify HMW glutenin subunits.

Forward PCR primers (5′………3′)	Reverse PCR primers (5′………3′)	References
P1 = 5′ ACCTTATCCATGCAAGCTACC 3′	P2 = 5′ CATGGCAGCCGACCGGCCAAC 3′	Smith et al. [22]
P3 = 5′ GTTGGCCGGTCGGCTGCCATG 3′	P4 = 5′ TGGAGAAGTTGGATAGTACC 3′	Smith et al. [22]
P5 = 5′ GCCTAGCAACCTTCACAATC 3′	P6 = 5′ GAAACCTGCTGCGGACAAG 3′	Ahmad [8]
P7 = 5′ AGCCTAGCAACCTTCAC 3′	P8 = 5′ AGACATGCAGCACATACC 3′	De Bustos et al. [15]
P9 = 5′ CTAACTCGCCGTGCACA 3′	P10 = 5′ AGCTAAGGTGCATGCATG 3′	De Bustos et al. [15]
P11 = 5′ CTCGTCCCTATAAAAGCCTAGT 3′	P12 = 5′ GAGACATGCAGCACATACT 3′	De Bustos et al. [15]
P13 = 5′ AGCTAAGGTGCATGCATG 3′	P14 = 5′ CTAACTCGCCGTGCACA 3′	De Bustos et al. [15]
P15 = 5′ ATGGCTAAGCGGTTAGTCCTC 3′	P16 = 5′ GCATTGTCGGCCAGCCAGTGA 3′	
P17 = 5′ GCATTGTCGGCCAGCCAGTGA 3′	P18 = 5′ ACAAGGGCAACAAGGTCAGCA 3′	
P19 = 5′ GCATTGTCGGCTAGCCAGTGA 3′	P20 = 5′ ATGGCTAAGCGGCTGGTCCTC 3′	

**Table 2:** PCR cycling information for amplifying specific alleles.

Primers	Annealing		Extension		Final extension	
	Temp	Time	Temp	Time	Temp	Time
P1P2	64°C	1′	72°C	1′	72°C	6′
P3P4	65°C	1′	72°C	1′	72°C	6′
P5P6	66°C	1′	72°C	1′	72°C	6′
P7P8	58°C	1′	70°C	2.20′′	70°C	7′
P9P10	66°C	1′	70°C	2.30′′	70°C	7′
P11P12	60°C	1.40′′	70°C	2.10′′	70°C	7′
P13P14	61°C	1′	70°C	2.30′′	70°C	7′
P15P16	61°C	1′	70°C	2′	72°C	7′
P17P18	59.5°C	1′	70°C	1′	70°C	7′
P19P20	62°C	50′′	70°C	2′	70°C	7′

**Table 3:** Comparing of protein mean by Duncan's multiple-range test.

Genotype	Mean protein percentage	Ranking
Ro-3	13.3	A
Roshan	12.6	B
Ro-4	12.3	B
Ro-5	12	C
Ro-1	11.8	C
Omid	10.6	D
O-64-10-10	10.6	D
Azar	10.2	E
T-65-9-1P	9.8	F
T-65-58-60	9.8	F
Tabasi	9.3	F
